# Factors influencing quality of life of people living with HIV in Estonia: a cross-sectional survey

**DOI:** 10.1186/1758-2652-12-13

**Published:** 2009-07-16

**Authors:** Kristi Rüütel, Heti Pisarev, Helle-Mai Loit, Anneli Uusküla

**Affiliations:** 1 Department of Illegal Drug Use and Infectious Diseases Prevention, National Institute for Health Development, Tallinn, Estonia; 2 Department of Public Health, University of Tartu, Tartu, Estonia; 3 Department of Chronic Diseases, National Institute for Health Development, Tallinn, Estonia

## Abstract

**Background:**

Identification of factors that determine quality of life is important in order to better tailor health and social care services, and thereby improve the functioning and well being of people living with HIV. The estimated number of people living with HIV in eastern Europe and central Asia is 1.6 million. Little is known about the quality of life of people living with HIV in this region. The main purpose of the present study was to identify the factors influencing quality of life in a sample of HIV-infected persons in Estonia.

**Methods:**

A convenient sample of 451 patients attending three infectious diseases clinics for routine HIV clinical care visits was recruited for a cross-sectional survey. The World Health Organization's Quality of Life HIV instrument was used to measure quality of life of the participants and medical data was abstracted from clinical records.

**Results:**

Good overall quality of life was reported by 42.6% (95% CI: 38.0–47.2%) of the study participants (53% men, 60% self-identify as injecting drug users, 82% <30 years of age, 30% with CD4+ T cell count <300 cells/mm^3^, and 22% on antiretroviral treatment). We identified the following variables as independent predictors of good overall quality of life: being currently employed or studying (AOR: 2.27, 95% CI: 1.18–4.38); and the absence of HIV-related symptoms (AOR: 2.31, 95% CI: 1.24–4.29).

**Conclusion:**

A comprehensive and competent care system, including health care providers and social workers, is required for an effective response. In addition, social interventions should seek to enhance the economic and employment opportunities for people living with HIV in the region.

## Introduction

A patient's well being is determined not only by his or her health status and response to treatment, but also by other social and psychological dimensions. The identification of factors that determine quality of life (QoL) is important in order to better tailor health and social care services, and thereby improve the functioning and well being of people living with HIV (PLHIV).

In addition, the identification of potentially modifiable factors of QoL could help target people in need of additional services in order to improve QoL [1]. Besides physical and mental health-related factors, socio-demographic characteristics, such as age, gender, education, income and employment status, have been found to be strongly associated with the QoL of PLHIV [2-4].

During the third decade of the AIDS era, new HIV epidemics continue to occur. One of the most recent and rapid increases in the number of new HIV infections in the world has taken place in the newly independent states of the former Soviet Union [5]. The estimated number of PLHIV in eastern Europe and central Asia is 1.6 million (1.2 million–2.1 million) [6]. In eastern Europe, injecting drug users (IDUs) accounted for 62% of all newly diagnosed HIV cases in 2006 [7]. Little is known about the QoL of PLHIV in this region.

The purpose of this study was to examine QoL among Estonian HIV-infected individuals, and to assess the impact of socio-demographic and disease-related variables on QoL in order to facilitate the development of treatment and social care programmes and interventions.

## Methods

### Setting and sample

In 2005, a cross-sectional survey was conducted using a convenient sample of HIV-infected patients attending for routine clinical care visits. We recruited study subjects from infectious diseases clinics in Tallinn, the capital of Estonia (N = 1), and in the north-eastern Ida-Viru county (N = 2). In total, 58% of the Estonian population live in the capital region and in north-eastern Estonia [8]. Importantly, approximately 90% of all HIV cases in Estonia are diagnosed in Tallinn and north-eastern Estonia [9] and, together, these three infectious diseases clinics serve more than 95% of all HIV-infected patients in care in Estonia [10].

These clinics provide both in-patient and out-patient infectious disease health care services, including HIV-specific services and antiretroviral (ARV) treatment. In each of the three clinics, one physician was contracted for the recruitment of study subjects, and was asked to enrol a minimum of 150 adult HIV-positive patients from an out-patient clinic.

Inclusion criteria for study subjects were that they be: more than 18 years old; have the ability to read and write in Estonian or Russian; and have been aware of their HIV-infection status for more than three months.

After determining eligibility and securing informed consent, all participants filled in the questionnaire designed for self-administration, which required between 45 and 60 minutes to complete. Participants received supermarket food vouchers to the value of approximately USD 10, equivalent to about 100 kroon (EEK), as an incentive for study participation.

### Measurements

A self-administered survey was used for socio-demographic, HIV-related risk behaviour and QoL assessment. Clinical characteristics were obtained from medical records.

Specifically:

1. QoL data was collected using the World Health Organization's Quality of Life HIV (WHOQOL-HIV) instrument. The WHOQOL-HIV contains 29 facets, each with four items, which are subsumed in six domains: physical, psychological, level of independence, social, environmental and spiritual. There is also a general facet that measures the overall QoL and general health perceptions (overall QoL). Items are rated on a 5-point Likert interval scale where 1 indicates low, negative perceptions, and 5 indicates high, positive perceptions. Facet scores are the mean of the four items in each facet. Domain scores are obtained by adding the facet means in the respective domain, dividing by the number of facets in that domain, and multiplying by 4, so that scores ranged from 4 (worst possible QoL) to 20 (best possible QoL). Adaptation of the WHOQOL-HIV instrument to the setting and to Estonian and Russian languages has been described elsewhere [11]. In short, Estonian and Russian versions of the WHOQOL-HIV proved to be reliable and valid instruments. Cronbach alpha was above 0.70 for the six domains and the overall QoL and general health facet. Each domain was significantly correlated with the overall QoL and general health facet. Asymptomatic people reported significantly better QoL in physical, psychological and independence domains and related facets compared to those people with symptoms or AIDS [[12]; Rüütel K, unpublished data].

2. We also collected data on socio-demographic characteristics (age, gender, education, marital status and employment status) and self-identified route of HIV-infection acquisition.

3. Physicians recruiting the patients collected clinical characteristics from hospital records on the basis of a standardized data abstraction form. Data on the stage of HIV infection (no symptoms, symptoms/early HIV disease, and AIDS), duration since diagnosis of HIV (3–6 months, 7–12 months, 1–2 years, 3–5 years, and more than 5 years), CD4 count, co-morbidities (hepatitis B and C, and tuberculosis) and antiretroviral treatment were obtained.

### Statistical analysis

Data entry was done centrally using Microsoft Access. Statistical analysis was performed with R 2.4.0, a language and environment software for statistical computing and graphics. We used proportions or means with standard deviation (for continuous data) to describe socio-demographic (age, region, gender, education and employment status) and health-related factors (disease stage, routes of infection, CD4 count and time since HIV diagnosis) in different QoL groups.

For the purposes of the factors of QoL analysis, participants were divided into two groups based on the mean score of the facet, "overall quality of life and general health perceptions" (range 1 to 5). Participants with mean scores of >3.0 were categorized as having good QoL, and their counterparts (mean scores of ≤ 3.0) as having poor QoL.

Odds ratios (OR) and 95% confidence intervals (95% CI), together with p-values, were used to identify variables associated with reporting good QoL. A multivariate analysis was performed using logistic regression, taking QoL (good/poor) as the binary dependent variable and the variables related to QoL in the univariate analyses as the covariates to evaluate the independent contribution of variables to QoL.

The magnitude of the association between covariates and QoL in univariate and multivariate analysis was evaluated through odds ratios, together with their corresponding 95% confidence intervals. P-values of less of 0.05 were considered as statistically significant.

Ethical approval was obtained from the Tallinn Medical Research Ethics Committee.

## Results

A total of 562 HIV-infected patients were approached for study participation between 1 June and 31 August 2005. Altogether, 451 (80%) were enrolled. The reasons for non-participation were as follows: refusal (50%, 56/111), being aware of HIV-infection status for less than three months (37%, 41/111), and being younger than 18 years at the time of the study (13%, 14/111). In all, 150 patients were recruited in the capital city, Tallinn (33%) and 301 in north-eastern Estonia (67%).

### Sample characteristics

Sample characteristics are presented in Table 1. The mean age of the participants was 25 years (SD 6.9 years), and 82% (n = 371) were younger than 30 years of age. More than half of the participants (53%, n = 240) were men. The majority of the participants were of Russian ethnicity (85%, n = 383); the remainder were either ethnic Estonians (10%, n = 45) or representatives of other nationalities (4.4%, n = 20). Close to two thirds (60%, n = 269) self-reported injection drug use (sharing needles) as a potential source of HIV infection; this included 33% of the women (n = 88) participating, and 67% of the men (n = 181). In total, 19% of the participants had been aware of their HIV infection for less than 12 months (n = 87), 24% for one to two years (n = 107), and 54% for more than three years (n = 245). In terms of HIV-related health status, 59% (n = 268) of the respondents were asymptomatic, 36% (n = 163) symptomatic, and 3% (n = 12) had AIDS.

**Table 1:** Socio-demographic, HIV disease and co-infection related characteristics of the participants

CHARACTERISTIC	N	%
*Socio-demographic*		

Gender		

Male	240	53.2

Female	210	46.6

N.A.	1	0.2

Age		

≥30 years	80	17.7

<30 years	371	82.3

Place of living		

Ida-Viru county, north-eastern Estonia	297	65.8

Capital city, Tallinn	132	29.3

Other	22	4.9

Ethnicity		

Russian	383	84.9

Estonian	45	10.0

Other	20	4.4

N.A.	3	0.7

Education		

≤9 years of formal education	180	39.9

>9 years of formal education	269	59.7

N.A.	2	0.4

Occupation		

Unemployed	161	35.7

Working or studying	284	63.0

N.A.	6	1.3

Partnership		

Legally married	106	23.5

Other	336	74.5

N.A.	9	2.0

*HIV disease related*		

HIV transmission category (self-report)		

Injecting drug use	269	59.7

Sexual transmission	163	36.1

Other	15	3.3

N.A.	4	0.9

Time of HIV diagnosis		

<12 months	87	19.3

1 to 2 years	107	23.7

≥3 years	245	54.3

N.A.	12	2.7

Stage of HIV-infection		

No symptoms	268	59.4

Early HIV disease	163	36.1

AIDS	12	2.7

N.A.	8	1.8

CD4 count		

≥300 cells/mm^3^	240	53.2

<300 cells/mm^3^	102	22.6

N.A.	109	24.2

Current ARV treatment		

Yes	97	21.5

No	352	78.0

N.A.	2	0.4

*Co-infections*		

Ever had tuberculosis		

Yes	10	2.2

No	439	97.4

N.A.	2	0.4

Ever had hepatitis B or C		

Yes	268	59.4

No	182	40.4

N.A.	1	0.2

N.A.: Data not available

### Quality of life

The mean scores for the overall QoL and general health and six domains for the sample are presented in Table 2.

**Table 2:** Mean scores for overall quality of life and general health perceptions and for six domains

	Mean score (SD)
Overall QoL and general health perceptions	2.9 (0.8)

Physical	13.3 (3.2)

Psychological	13.7 (2.3)

Independence	14.5 (3.2)

Social relationships	13.9 (2.6)

Environment	12.3 (2.1)

Spiritual	12.8 (2.9)

In a univariate analysis, the factors which significantly increased the likelihood of good QoL in the facet, "overall QoL and general health perceptions", included: female gender (48% vs 37% among males, p = 0.03); age under 30 years (45% vs 29%, p = 0.009); living in the capital city (57% vs 36% in Ida-Viru county, p = 0.0001); being employed or studying (52% vs 38% among unemployed, p < 0.0001); being legally married (55% vs 39% among people in other types of relationships, p = 0.004); being infected with HIV sexually, not through injecting drug use, based on self-report on the mode of HIV acquisition (53% vs 38%, p = 0.002); being aware of their infection for less than 12 months (54% vs 41%, p = 0.02); having no HIV-related symptoms, based on abstraction from clinical records (51% vs 28%, p < 0.0001); and CD4 count above 300 cells/mm^3^, based on abstraction from clinical records (54% vs 32%, p = 0.0003) (Table 3).

**Table 3:** Univariate and multivariate factors associated with good quality of life among HIV-infected persons in Estonia

Characteristic	Good QoL		OR	95% CI	p	AOR	95% CI	p
							
	N	%						
*Socio-demographic*								

Gender								

Male	89	37.4	1.0			1.0		

Female	100	47.6	1.52	1.04–2.22	0.03	0.61	0.33–1.13	0.1

Age								

≥30 years	23	28.7	1.0			1.0		

<30 years	166	45.0	2.03	1.20–3.43	0.009	1.55	0.74–3.25	0.2

Place of living								

Ida-Viru county, north-eastern Estonia	105	35.5	1.0			1.0		

Capital city, Tallinn	74	56.5	2.4	1.56–3.57	0.0001	1.08	0.62–1.88	0.8

Ethnicity								

Russian	157	42.3	1.0					

Estonian	24	55.8	1.7	0.91–3.25	0.09			

Education								

≤9 years	69	38.3	1.0					

>9 years	120	44.9	1.31	0.89–1.93	0.2			

Occupation								

Unemployed	123	38.2	1.0			1.0		

Employed and/or studying	65	52.0	3.13	2.04–4.76	<0.0001	2.27	1.18–4.38	0.01

Partnership								

Other	129	38.6	1.0			1.0		

Legally married	58	54.7	1.92	1.24–2.99	0.004	1.26	0.66–2.41	0.5

*HIV disease related*								

HIV transmission category (self report)								

Injecting drug use	89	37.7	1.0			1.0		

Sexual transmission	86	53.4	1.89	1.27–2.86	0.002	1.37	0.73–2.59	0.3

Time of HIV diagnosis								

≥12 months ago	99	40.6	1.0			1.0		

<12 months ago	47	54.0	1.79	1.1–2.86	0.02	1.2	0.60–2.45	0.6

Stage of HIV-infection								

Early HIV disease/AIDS	49	28.2	1.0			1.0		

No symptoms	136	50.9	2.65	1.76–3.98	<0.0001	2.31	1.24–4.29	0.008

CD4 count								

<300 cells/mm^3^	32	31.7	1			1.0		

≥300 cells/mm^3^	128	53.6	2.49	1.52–4.06	0.0003	1.72	0.91–3.26	0.09

Current ARV treatment								

No	136	40.2	1.0					

Yes	52	48.1	1.37	0.89–2.13	0.1			

*Co-infections*								

Tuberculosis								

Yes	4	40.0	1					

No	184	42.1	1.09	0.3–3.92	0.9			

Hepatitis B and/or C co-infection								

Yes	106	39.8	1					

No	83	45.6	1.27	0.86–1.85	0.2			

In multivariate analysis (logistic regression model), after including variables significant in univariate analysis into the model, being currently employed or studying (AOR: 2.27, 95% CI: 1.18–4.38), and the absence of HIV-related symptoms (AOR: 2.31, 95% CI: 1.24–4.29) were identified as independent predictors of good QoL (Table 3).

## Discussion

This is the first study describing factors influencing QoL of HIV-infected persons from an eastern European country, a region that has witnessed a relatively recent HIV epidemic driven by injection drug use. It has been suggested that transmission of 80% of HIV infections in former Soviet Union countries is attributable to sharing needles and syringes [7].

The mean overall QoL score for the whole sample (2.90 ± 0.84) was slightly lower than that reported from similar studies from other regions. O'Connell and colleagues have described a mean overall QoL score (measured by WHOQOL) of 3.2 ± 0.88 for a sample of 590 HIV-infected persons from six culturally diverse sites in Australia, Brazil, India (two sites), Thailand and Zimbabwe [13].

Further, we can argue that our study overestimated the QoL of PLHIV in Estonia. Several studies have documented better physical health-related QoL among former IDUs than in current IDUs [14,15]. IDUs often struggle with multiple health risks due to social, economic and psychological factors. Getting HIV care may not be their top concern because they face other more pressing daily challenges, such as addiction, poverty, incarceration, homelessness, depression, mental illness and past trauma [16].

Given this and the highly stigmatised nature of illegal drug use, both former and current injecting drug users are less likely to receive HIV-related medical care – this is the case in Estonia – and hence they may be under-represented in the clinical samples of PLHIV. In our sample, 60% of participants reported having used injecting drugs.

In our study, the independent and most influential contributors to the general QoL were a person's employment status and clinician-recorded HIV-disease stage. The employed participants, including those who were studying, were more likely to have good general QoL than their not-working counterparts. Our findings are consistent with previous research, which has demonstrated that employment (and higher income) is associated with a better QOL among PLHIV [17,18].

Controlling for disease severity, employed individuals report significantly higher level of perceived QoL than those who are unemployed [19-21]. Besides financial benefits, employment also provides a source for structure, social support, role identity and meaning. In addition, stable income and employment have been associated with adherence to highly active antiretroviral treatment [22].

Employment may also provide resources, which buffer the effects of the stress of HIV infection and thus serves to maintain a sense of quality of life [21]. Therefore, return-to-work programmes and other interventions to enhance the economic and employment opportunities are important for PLHIV in eastern Europe and the Russian Federation. Here, young injecting drug users are the main HIV risk group, and unemployment among them is high.

According to our results, asymptomatic patients reported better QoL than those with symptoms or AIDS diagnosis. This factor is amenable for clinical interventions. Quality of life can be altered by both the immediate effects and the longer-term consequences of antiretroviral treatment. Persons with advanced HIV disease and low QoL scores have demonstrated significant improvements in QoL with ARV treatment [23,24].

Other disease-related factors and co-infection with hepatitis B or C were not associated with better QoL in our sample. Nevertheless, given the extremely high (>90%) hepatitis C infection rates among IDUs in the region and the high proportion of IDUs among PLHIV [25,26], co-infections with hepatitis B or C warrant attention. Several previous studies have suggested that hepatitis C infection significantly reduces health-related QoL [27,28], and this effect can be inversed with antiviral treatment [29]. Chronic hepatitis B infection similarly negatively impacts on QoL [30].

With improvements in HIV treatment, liver disease has become a major cause of hospitalisation and death in PLHIV and complications related to hepatitis B or C co-infection are becoming an increasingly important medical issue. Proper prevention, screening and management of co-infections are of great concern given the high rates of hepatitis B and C infections in Estonia.

### Limitations

Our study had limitations. Given the design of the study, we are able to demonstrate neither causality nor the direction of the described associations. The degree to which the study is representative of the larger HIV-infected population is influenced by the potential selective factors associated with recruiting from HIV treatment settings.

Our results most likely overestimate the QoL of those infected with HIV, as we have discussed. We were successful in recruiting people who are in HIV care in Estonia by sampling from the facilities that provide 95% of HIV care in the country. We also achieved a high participation rate in a specified study period.

To decrease the potential for social desirability bias, we used a self-administered survey instrument for risk behaviour data collection. To enhance the validity of the data on health status, we complemented self-reports with clinical data abstracted from clinical records.

## Conclusion

In conclusion, HIV disease status and employment were the important factors influencing QoL of PLHIV in Estonia. A comprehensive and competent care system, including medical staff and social workers, is required for an effective response. In addition, social interventions should seek to enhance the economic and employment opportunities for PLHIV.

This data contributes to the input needed for planning health care services and interventions that address QoL improvement for PLHIV in eastern Europe and central Asia.

## Competing interests

The content of this paper has not been published elsewhere; nor is it being considered for publication elsewhere. The authors declare that they have no competing interests.

## Authors' contributions

KR and HML designed the study. HML supervised the data collection. KR, AU and HP designed the data analysis and structure of the manuscript. HP conducted the statistical analysis. KR wrote the first draft of the manuscript. All of the authors contributed to the final version of the manuscript.
